# ﻿Phylogenomics and a new classification of the tropical genus *Heliconia* L. (Monocots, Zingiberales, Heliconiaceae)

**DOI:** 10.3897/phytokeys.251.130409

**Published:** 2025-01-13

**Authors:** W. John Kress, Tomáš Fér, Mónica M. Carlsen

**Affiliations:** 1 Department of Botany, United States National Herbarium, National Museum of Natural History, Smithsonian Institution, P.O. Box 37012, Washington, DC 20013-7012, USA Smithsonian Institution Washington United States of America; 2 Department of Botany, Faculty of Science, Charles University, Benátská 2, CZ 12800, Prague, Czech Republic Charles University Prague Czech Republic; 3 Science & Conservation Division, Missouri Botanical Garden, 4344 Shaw Boulevard, St. Louis, MO 63110, USA Missouri Botanical Garden St. Louis United States of America

**Keywords:** Classification, phylogenomics, target enrichment, tropical, Zingiberales bait set

## Abstract

Members of the genus *Heliconia* L. (Heliconiaceae) have evolved complex interactions with both insect herbivores and hummingbird pollinators in tropical forests and secondary growth where they are abundant and diverse. Many of these same species have also been cultivated as ornamentals around the world for hundreds of years because of their extraordinary colors and forms. Because of the large size, fleshy nature, and tropical distribution, and despite a long taxonomic history, the classification and phylogenetic relationships of species of *Heliconia* have not received sufficient attention to date. No complete classification has been published for the entire genus, although some preliminary attempts have been offered. In this paper we used tissue sampled from field and herbarium collections of 136 species for genomic sequencing to determine the phylogenetic patterns within *Heliconia*, which then served as the basis for a new evolutionary classification of the genus. This new classification, which is based on extensive field work and the phylogenomic insights provided here, includes 187 currently recognized species. The new classification of *Heliconia* is composed of 17 sections in five subgenera with all groups well-supported in the phylogenomic analysis. Four subgenera are each composed of two sections and one subgenus includes nine sections. One subgenus and 10 sections are described as new.

## ﻿Introduction

### ﻿The Genus *Heliconia* L. – background

The Zingiberales, an order of monocots native almost exclusively to tropical habitats, are comprised of eight families, 110 genera, and over 2,600 species (Carlsen et al. 2018). Many of these species are of economic and cultural importance to humans, including major agricultural crops (e.g., bananas, in the genus *Musa*), culinary staples (e.g., turmeric and ginger, in the genera *Curcuma* and *Zingiber*), horticultural ornamentals (e.g., prayer plants [*Calathea*], globbas [*Globba*], cannas [*Canna*], and heliconias [*Heliconia*]), and even national symbols (e.g., the travelers tree of Madagascar, in the genus *Ravenala* Adans.).

Members of the genus *Heliconia* (Heliconiaceae) have been cultivated as ornamentals around the world for hundreds of years because of their extraordinary colors and forms, and continue to have a prominent position in the horticultural trade (Berry and Kress 1991). A professional society (www.heliconia.org) is devoted to consolidating and distributing information on these plants to scientists, amateur enthusiasts, and the commercial trade community. Many of these same species as well as others in the genus have also been shown to have evolved complex interactions with both insect herbivores (e.g., Garcia-Robledo et al. 2013a, b) and hummingbird pollinators (e.g., Temeles and Kress 2003; Temeles et al. 2012; Gowda and Kress 2013) in tropical forests and secondary growth where they are abundant and diverse.

An understanding and appreciation of the evolutionary history of *Heliconia* as well as species delimitations and classification will assist scientists in determining the breadth of ecological patterns in community composition, help horticulturists to promote commercial utilization of these plants, and inform conservationists in determining workable plans to protect species. Unfortunately, because of their large size, fleshy nature, and tropical distribution, the taxonomy and phylogenetic relationships of the 187 currently recognized species of *Heliconia*, have not received sufficient attention until now to fully understand their evolutionary relationships. Moreover, no complete classification has been published for the entire genus, although some preliminary attempts have been offered (e.g., Andersson 1992; Kress et al. 1999). Here, a phylogenomic analysis of species relationships coupled with extensive field work provide the foundation for a revised classification of *Heliconia*.

### ﻿Taxonomy and classification – a history

The first botanical description of *Heliconia* was by Plumier (1703) in “Nova Plantarum Americanarum Genera”. He provided a short generic description of the genus *Bihai* and polynomials for three taxa. In “Species Plantarum” Linnaeus (1753) combined these three taxa into a single species, *Musabihai*, retaining Plumier’s exact diagnoses and placing the “variegatis” variety first. Miller (1754, 1768) and Adanson (1763) considered these plants generically distinct from other species of *Musa* and used the name *Bihai*. In “Mantissa Plantarum”, Linnaeus (1771) also segregated *M.bihai* into its own genus, *Heliconia* L., and provided a generic description with a short description of a single species, *H.bihai*. Kuntze (1891; substituting the variant spelling *Bihaia*) and later Griggs (1904, 1915) recognized the earlier generic name and transferred all species of *Heliconia* known to them into *Bihai*. However, at the International Botanical Congress held in Vienna in 1905, *Heliconia* was reinstated as a *nomen conservandum* (Farr et al. 1979).

Around the turn of the last century, a number of workers attempted revisions or summaries of the genus, including Petersen (1890), Kuntze (1891), Baker (1893), and Schumann (1900). Griggs, one of the most knowledgeable students of *Heliconia* due to his study of plants in the field, subsequently published several papers on the genus (1903, 1904, 1915), eventually accepting 38 neotropical species. In the latter decades of the twentieth century a flurry of new species was recognized and described (e.g., Daniels and Stiles 1979; Abalo and Morales1982, 1983; Kress 1984; Andersson 1985a) and today 187 species are accepted here with many names in synonymy.

With regards to infrageneric classification of *Heliconia*, early efforts were based solely on the shape of the cincinnal bracts with later workers adding plant size, leaf orientation, and inflorescence habit and structure to devise more detailed classifications. Kuntze (1891) published the first infrageneric taxon (sect. Taeniostrobus Kuntze) above the rank of species. Baker (1893) later divided the genus into two subgenera, *Platychlamys* Baker and *Stenochlamys* Baker, recognizing 29 species in all (although some of these species are no longer accepted today). Schumann (1900) followed Baker’s classification except he placed *Platychlamys* in synonymy under *Taeniostrobus* and altered the ranks of these taxa from subgenera to sections.

Griggs (1903) recognized that infrageneric groups based upon a single character, for example cincinnal bract shape, were inadequate. He subsequently used plant habit and cincinnal-bract orientation to build a diagnostic key that included three subgenera, *Stenochlamys*, *Platychlamys*, and *Taeniostrobus*, which had been earlier defined by others. However, in his final publication (Griggs 1915) he replaced this classification with one consisting of two subgenera (*Taeniostrobus* and *Stenochlamys*) and six subordinate taxa of unspecified rank. Plant habit and height, inflorescence orientation, and distance between adjacent inflorescence bracts were used to recognize subgeneric groups and all 38 neotropical species known at that time were included in his treatment.

No attempts were made to revise Griggs’ classification until the latter part of the twentieth century with publications by Andersson (1981, 1985a, b, 1992), Kress (1984, 1990), Kress et al. (1993), Betancur and Kress (1995), and Kress et al. (1999). Andersson used a wider suite of morphological traits (including flower resupination and pollen features) and in his latter publications based his classifications on a cladistic analyses of these characters. His first paper (Andersson 1981) deals with the circumscription of Heliconiasect.Heliconia; the second treatment (Andersson 1985a) subdivided Heliconiasubgen.Stenochlamys into six sections (*Lanea*, *Stenochlamys*, *Proximochlamys*, *Lasia*, *Cannastrum*, and *Zingiberastrum*); and in the third publication (Andersson 1985b), species were arranged into four subgenera (*Taeniostrobus*, *Heliconia*, *Stenochlamys*, and *Griggsia*), with several subgenera further divided into both formal and informal groups. In his final publication Andersson (1992) provided the most complete and detailed classification of the genus to date recognizing five subgenera, ten sections, and eight informal groups (subgen. Taeniostrobus; subgen. Heliconia [sects. *Episcopalis*, *Heliconia*, *Tortex*, *Tenebria*]; subgen. Stenochlamys [sects. *Lanea*, *Stenochlamys*, *Proximochlamys*, *Lasia*, *Cannastrum*, *Zingiberastrum*]; subgen Heliconiopsis ; subgen. Griggsia [informal groups *H.griggsiana*, *H.pogonantha*, *H.longa*, *H.platystachys*, *H.rostrata*, *H.trichocarpa*, *H.obscura*, and *H.nutans*]). Some of the informal groups established by Andersson within subgenus Griggsia had been earlier designated by Kress (1984).

Subgenus Heliconiopsis, encompassing the tropical Asian-Pacific species of the genus, has been an anomaly treated quite differently by various authors. Miquel (1859) erected the new genus *Heliconiopsis* for the only Asian-Pacific species known at that time. Baker (1893), Schumann (1900), and Winkler (1930) synonymized all taxa proposed for Asian-Pacific material under *Heliconiabihai*. Green (1969) later recognized that tropical Asian-Pacific heliconias were quite different from those in the American tropics, but considered that all populations belonged to a single polymorphic species, *Heliconiaindica* Lam. Eventually, after extensive field work, Kress (1990) recognized six species that stretched from Samoa to Papua New Guinea and New Caledonia. He, as concurred by Andersson, considered these species to constitute a diagnosable group recognized as subgen. Heliconiopsis.

Most recently, Kress and colleagues (Kress et al. 1993; Betancur and Kress 1995; Kress et al. 1999) have proposed a series of preliminary classifications that were built on earlier taxonomies and based primarily on morphological traits. These newer classifications were a response in part to the many new species of *Heliconia* discovered and described from Costa Rica, Panama, Colombia, Ecuador, and Peru in the 1980s and 1990s. These categorizations were an attempt to account for the massive diversity represented by these new taxa and often focused on species from a single country. The most comprehensive preliminary classification (Kress et al. 1999) encompassed the 93 species then known from Colombia and included four subgenera and 22 sections, many indicated as “ined.” by the authors to denote that the work was in progress (subgen. Heliconia [sects. *Heliconia*, *Episcopalis*, *Tortex*, *Farinosae* “ined.”, *Complanatae* “ined.”, and *Tenebria*]; subgen. Taeniostrobus ; subgen. Stenochlamys [sects. *Lanea*, *Stenochlamys*, *Proximochlamys*, *Lasia*, *Cannastrum*, and *Zingiberastrum*], subgen. Griggsia [sects. *Griggsia* “ined.”, *Barbatae* “ined.”, *Arcuatae* “ined.”, *Longae* “ined.”, *Obscurae* “ined.”, *Dromedarius* “ined.”, *Sigmoideae* “ined.”, *Rostratae* “ined.”, *Pendulae* “ined.”, and *Retiformes*, “ined.”]). No species in subgen. Heliconiopsis were included as none are known from South America.

The only molecular analysis of phylogenetic relationships in *Heliconia* was provided by Iles et al. (2017). They demonstrated that subgenera and sections designated by earlier taxonomists as outlined above (e.g., Andersson 1992; Kress et al. 1993; Kress et al. 1999) were not monophyletic. However, with the molecular data in their analysis limited to seven plastid and nuclear markers, the support values for almost all clades throughout the tree were very low. None-the-less, this earlier analysis has provided a gene-based foundation for the more extensive genomic-based analysis in the current investigation.

## ﻿Materials and methods

### ﻿Taxon sampling

We included 136 *Heliconia* species (ca. 73% of the species in the genus) representing the entire spectrum of morphological and geographical diversity within the genus, as well as 5 outgroup genera (Musaceae: *Ensetesuperbum* (Roxb.) Cheesman and *Musellalasiocarpa* (Franch.) C.Y. Wu; Lowiaceae: *Orchidanthachinensis* T.L. Wu; and Strelitziaceae: *Strelitzianicolai* Regel & Körn. and *Ravenalamadagascariensis* Sonn.). See Suppl. material 1: table S1 for specimen information and accession numbers.

### ﻿Library preparation and target loci capture

Total genomic DNA was extracted from either silica dried leaves or herbarium specimens using a modified CTAB protocol (Doyle and Doyle 1987). Libraries were constructed using the NEBNext Ultra™ II DNA Library Prep kit with Sample Purification Beads and Multiplex oligos for Illumina (96 Index Primes) (New England BioLabs Inc., Ipswich, MA, USA). Library concentration and expected size were confirmed using a High Sensitivity D1000 ScreenTape run on a 4150 TapeStation system (Agilent Technologies Inc., Santa Clara, CA, USA), and Qubit dsDNA HS Assay Kit run on a Qubit 2.0 Fluorometer (Invitrogen, Carlsbad, CA, USA). Libraries were combined in hybridization pool reactions consisting of 10–12 *Heliconia* species per reaction. All these steps were performed at the Laboratories for Analytical Biology (LAB) of the Smithsonian National Museum of Natural History. In solution hybridization of the library pools to the target loci was performed by Arbor Biosciences LLC (Ann Arbor, Michigan) using the myBaits Custom 1-20K kit designed for Zingiberales (Carlsen et al. 2018). Sequencing was performed either in an illumina MiSeq with v.3 chemistry kits (250 bp paired-end) at the Smithsonian or in an illumina NovaSeq S4 system (150 bp paired-end) by Arbor Biosciences (Illumina Inc., San Diego, CA).

### ﻿Bioinformatic analyses

Raw paired-end reads were quality trimmed and adapters were removed using a combination of Trimmomatic v. 0.39 (Bolger et al. 2014) with the following parameters ILLUMINACLIP:TruSeq3-PE-2NEB.fa:2:30:10 LEADING:3 TRAILING:3 SLIDINGWINDOW:10:20 MINLEN:40 and Trim Galore v. 0.6.4 (https://github.com/FelixKrueger/TrimGalore) with default parameters. Cleaned reads were assembled using HybPiper v. 1.3.1 (Johnson et al. 2016). Coding sequences were extracted using Exonerate (Slater and Birney 2005) with the HybPiper retrieve_sequences.py script. From the set of fasta files resulting for each locus, we removed: 1. Loci with no data, 2. Loci containing species with extremely long branches (i.e., loci matched is 150% longer than reference loci), 3. Loci with paralogs warnings in HybPiper, 4. Loci without a single outgroup taxa represented, and 5. Loci recovered for less than 75% of the taxa of *Heliconia* sampled (i.e. with fewer than 102 taxa of *Heliconia* represented). Each of the remaining loci recovered were separately aligned with MAFFT v. 7.407 (Katoh and Standley 2013) with “--auto” parameter. Alignments were manually checked for misaligned regions and extremely gappy areas, which were either removed or fixed. Alignment statistics were calculated with AMAS (Borowiec 2016), and from the results of this analysis, we further removed each individual taxa with >50% missing sequence for a given locus and any individual locus with >50% total missing data per alignment. Single locus tree reconstruction was performed using RAxML v. 8.2.12 (Stamatakis 2014), with no partition scheme, GTRCAT model of evolution, and 500 bootstrap replicates. Coalescence based species tree reconstruction was performed with ASTRAL III v. 5.7.1 (Zhang et al. 2018) using the individual trees generated with RAxML above. Individual branches with low support in these trees were collapsed if bootstrap support was below 50%. All bioinformatics analyses were performed in the Smithsonian Institution High Performance Computing Cluster (SI HPC – Hydra) (https://doi.org/10.25572/SIHPC).

### ﻿Constructing the revised classification

The resultant *Heliconia* species tree topology was assessed for congruence with past classifications and for major well-supported clades corresponding to diagnostic morphological traits and geographic patterns. Groups of species, which had been assigned to subgenera and sections in the earlier classifications, were assessed with respect to the newly identified clustering of taxa and clade support. Only clades with Local Posterior Probability (LPP) 0.90 or greater were considered for higher ranks at the subgeneric and sectional level.

In this phylogenomic analysis molecular data were only available for 136 of the 187 currently recognized species of *Heliconia*. Therefore, the remaining 51 species were placed into subgenera and sections according to their morphological characteristics only. In the case of significant conflict between the phylogenetic placement and morphological traits, taxa were tentatively classified according to their morphological characteristics. Their placement will be tested in future analyses with additional field observations and molecular data.

### ﻿Geographic distribution

Distributions of species by country within each subgenus and section were determined from specimen data included in an analysis of conservation assessment (Kress et al. 2024) and as part of a taxonomic monograph of the genus (Kress unpubl.).

## ﻿Results and discussion

### ﻿A new genome-scale dataset for *Heliconia*

All raw reads newly generated for this study are deposited in NCBI BioProject accession number PRJNA1204471. The average number of trimmed, high-quality, non-duplicated reads obtained was 3,242,087 per *Heliconia* sample, ranging from 368,676 reads (in *H.marginata* (Griggs) Pittier) to 19,241,471 reads (in *H.adflexa* (Griggs) Standl.) (Suppl. material 1: table S1). On average 51% of these high-quality reads mapped to the target nuclear loci from the Zingiberales bait set (Carlsen et al. 2018), ranging from 19% (in *H.lingulata* Ruiz & Pav.) to 75% (in *H.trichocarpa* Daniels & Stiles). A final set of 452 high-quality target nuclear loci was used for phylogenetic tree inference after a strict and comprehensive data clean-up (see Methods above). They represent ca. 38% of the total 1,180 target loci included in the Zingiberales bait set. The number of high-quality target loci recovered for individual *Heliconia* species ranged from 275 in *H.marginata* to 448 in *H.carmelae* Abalo & Morales. The alignment length of each individual high-quality nuclear locus ranged from 300 to 3,496 bp (on average 1,002 bp), their total amount of missing data from 0 to 49% (on average 17%), and the proportion of informative sites varied between 0.03 and 0.48 (on average 0.14) (Suppl. material 1: table S1). Individual loci alignments are available on Dryad [https://doi.org/10.5061/dryad.mcvdnck9g].

**Figure 1.**
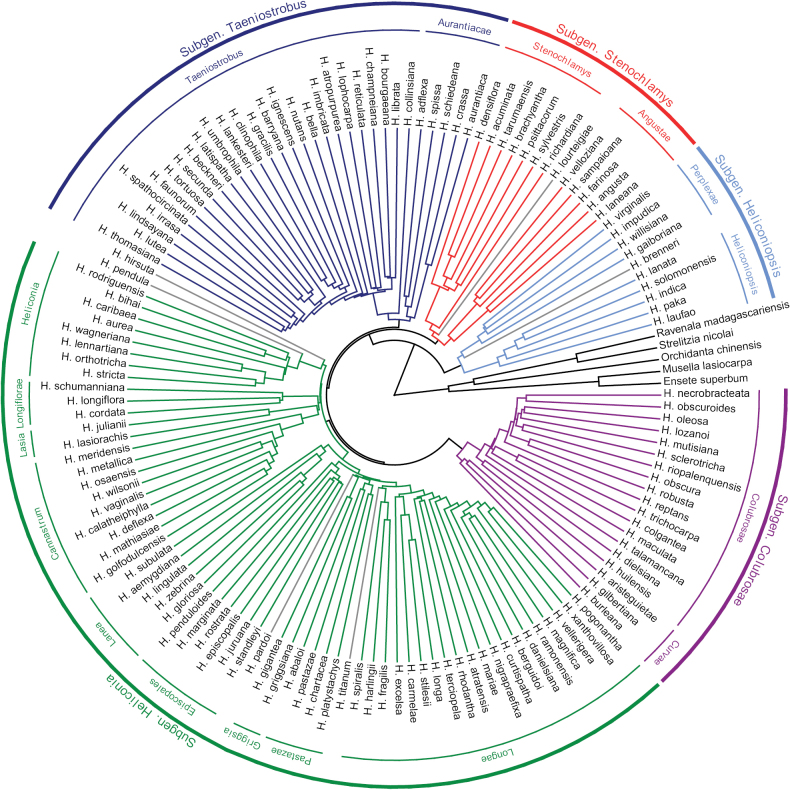
Circular phylogeny of species of *Heliconia* included in the genomic analysis with the new classification indicated. The six species with gray branches represent significant conflicts in genomic and morphological evidence, with the latter given priority in species placement in the classification (see text and Suppl. material 2: fig. S1).

The topology recovered in the coalescence species tree analysis using ASTRAL (Fig. 1) shows high LPP support values of 0.90–1.00 for most branches (Suppl. material 2: fig. S1). Only nine branches along the backbone of the *Heliconia* phylogeny were weakly supported by 0.46–0.89 LPP; these are among the shortest branches of the tree. Some shallow branches (i.e. species-pairs relationships) with LPP support values lower than 0.90 are scattered throughout the phylogeny (Suppl. material 2: fig. S1).

### ﻿A new genome-based phylogeny of *Heliconia*

**Figure 2.**
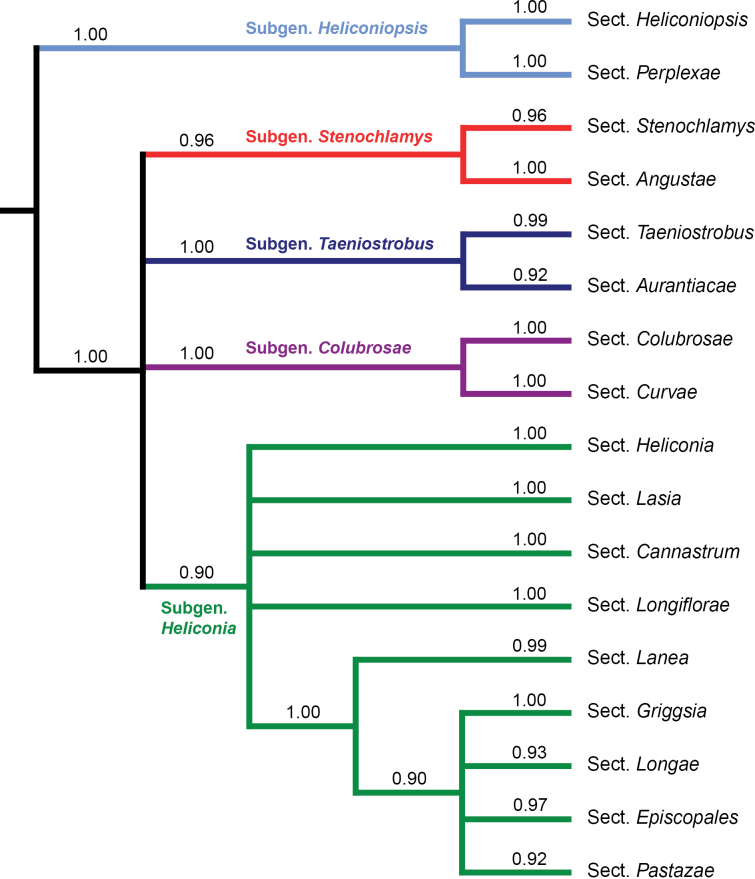
Synopsis of the new classification based on the genomic analysis (Local Posterior Probability [LPP] is indicated for each branch).

The phylogenomic analysis of nearly three-quarters of the known species of *Heliconia* provides a robust evolutionary framework and a firm foundation for a newly revised classification of the genus (Figs 1, 2; see below). All five major lineages (i.e., designated here as taxonomic subgenera; Fig. 2) in the backbone of the new phylogeny of *Heliconia* and all 17 clades within them (i.e., designated here as taxonomic sections; Fig. 1) are highly supported, even though relationships among some of the subgenera and sections are not fully resolved (Figs 1, 2). This is a striking difference when compared to the previous phylogeny of *Heliconia* (Iles et al. 2017), in which the bootstrap support value for almost all clades was very low. Of the major lineages recognized here, only one (subgenus Heliconiopsis) had bootstrap support greater than 90% in Iles et al. (2017). Two of the remaining four major lineages recovered here (subgenera *Stenochlamys* and *Heliconia*) were polyphyletic in their earlier analysis, whereas the other two (subgenera *Colubrosae* and *Taeniostrobus*) had only weak bootstrap support (<70%). At the sectional level, only six of the sections recognized in our analyses (sections *Heliconiopsis*, *Perplexa*, *Stenochlamys*, *Lasia*, *Longiflorae* and *Lanea*) were supported by bootstrap values greater than 90% in their analysis. For the remaining eleven sections, most of the species recognized here were clustered together in the Iles et al. (2017) analysis, but almost all of these sections lacked even moderate statistical support. Clearly more nucleotide data were needed to resolve their basic cladistic structure than were available in that investigation. Iles et al. (2017) concluded that subgenera and sections designated by earlier taxonomists (e.g., Andersson 1992; Kress et al. 1993; Kress et al. 1999) were not monophyletic, but the authors did not propose a new classification, perhaps due to the lack of support for most clades recovered in that study.

The new classification of the 187 currently recognized species of *Heliconia* presented below takes into consideration earlier suggested classifications, but more importantly utilizes the newly generated extensive phylogenomic data set to support the proposed taxonomic groups. Additionally, most of the 51 species for which genomic data were not available have also been confidently placed in the classification based on their morphological characteristics. Only six species with molecular data proved difficult to classify because of substantial disparities between their placement in the phylogeny and their morphological characteristics. These taxa (*H.hirsuta*, *H.lourteigiae*, *H.pendula*, *H.pardoi*, *H.brenneri*, and *H.titanum*; indicated by “†” in the classification below) were tentatively classified according to their morphological characteristics. In the phylogeny recovered here, all of these taxa are positioned towards the base of their respective clades, therefore suggesting that these disparities may be due to sampling or sequencing errors and further analyses are needed to verify their placements.

More data are clearly needed to confidently place the few species with genomic data but contradictory morphological characters as well as verifying the position of the taxa lacking any genomic data. As new molecular traits become available and more detailed observations of living plants are conducted in the field in both Central and South America, it is expected that some species will shift position in the classification and that new taxa will be added as they are discovered and described. As detailed in a companion publication (Kress et al. 2024), increased environmental degradation and overexploitation of some taxa may significantly limit the time remaining for this future work without significant efforts in the protection of populations, species, and habitats.

### ﻿A new classification of *Heliconia*

**Figure 3.**
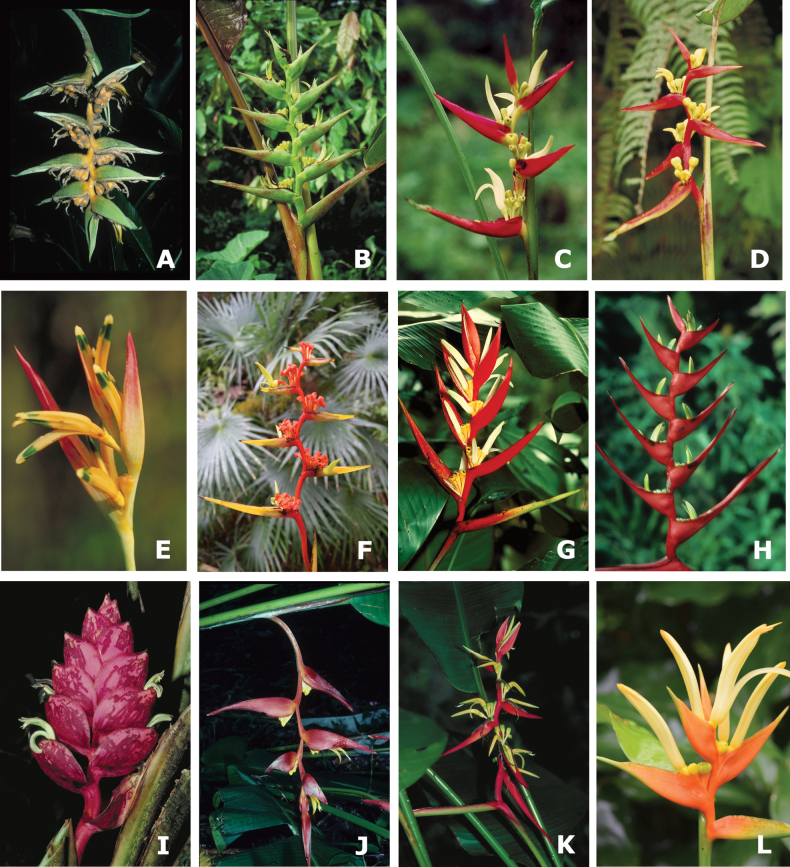
Representatives of three subgenera and six sections in the new classification of *Heliconia***A–D** subgen. Heliconiopsis**A, B** sect. Heliconiopsis: **A***H.solomonensis***B***H.indica***C, D** sect. Perplexae: **C***H.gaiboriana***D***H.impudica***E–H** subgen. Stenochlamys**E, F** sect. Stenochlayms: **E***H.psittacorum***F***H.richardiana***G, H** sect. Angustae: **G***H.laneana***H***H.velloziana***I–L** subgen. Taeniostrobus**I, J** sect. Taeniostrobus: **I***H.reticulata***J***H.collinsiana***K, L** sect. Aurantiacae: **K***H.schiedeana***L***H.aurantiaca*.

**Figure 4.**
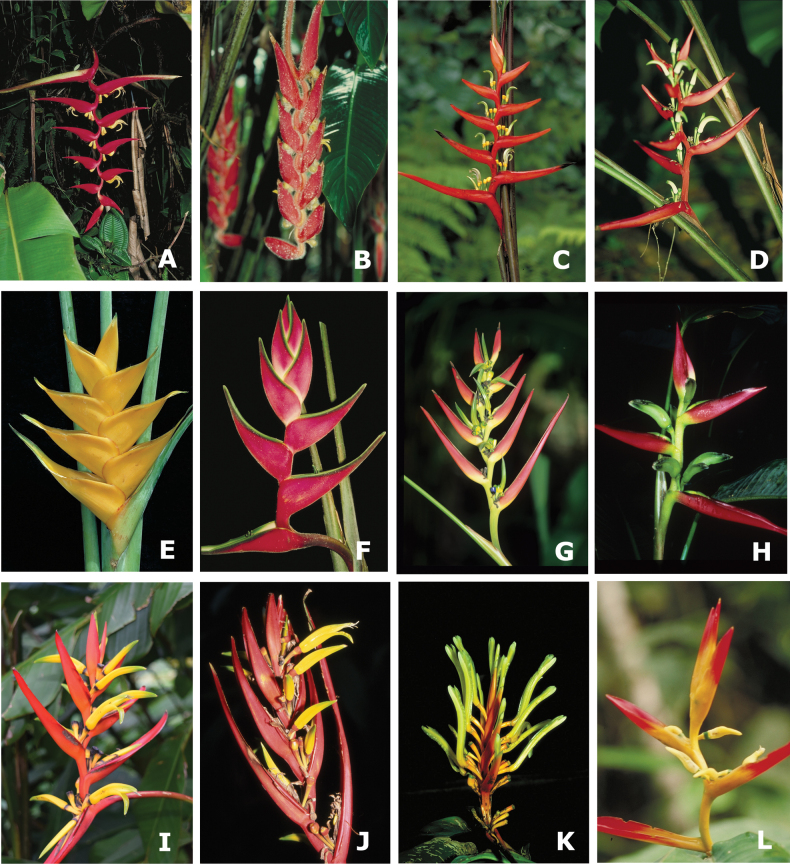
Representatives of two subgenera and six sections in the new classification of *Heliconia***A–D** subgenus Colubrosae**A, B** sect. Colubrosae: **A***H.dielsiana***B***H.mutisiana***C, D** sect. Curvae: **C***H.burleana***D***H.gilbertiana***E–L** subgen. Heliconia**E, F** sect. Heliconia: **E***H.bihai***F***H.orthotricha***G, H** sect. Lasia: **G***H.julianii***H***H.lasiorachis***I, J** sect. Cannastrum: **I***H.deflexa***J***H.venusta***K, L** sect. Longiflorae: **K***H.longiflora***L***H.schumanniana*.

**Figure 5.**
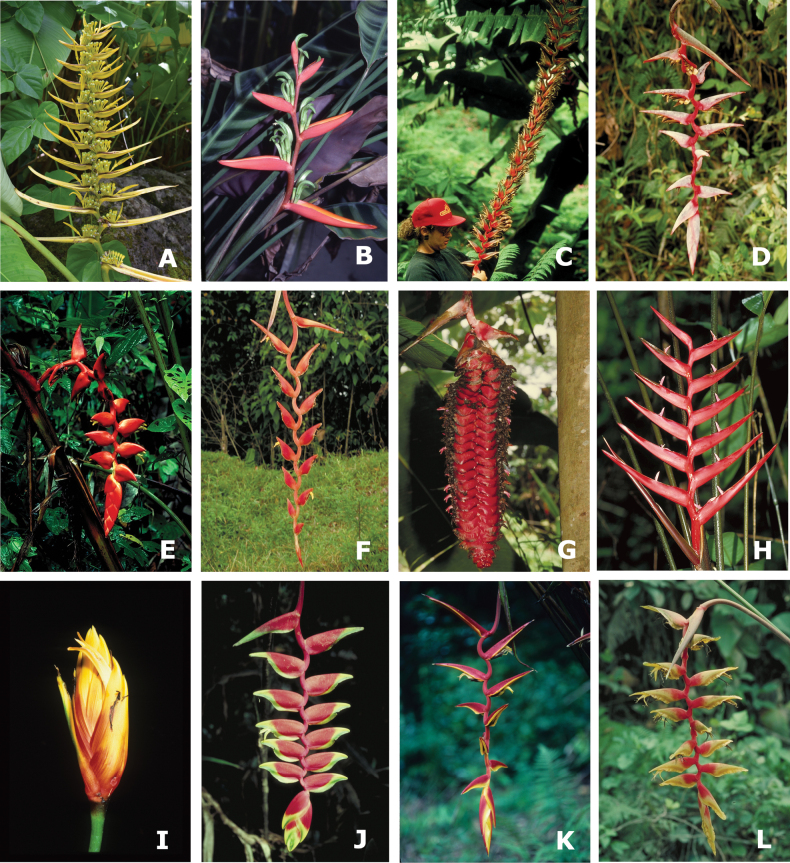
Representatives of one subgenus and five sections in the new classification of *Heliconia***A–L** subgenus Heliconia**A, B** sect. Lanea: **A***H.lingulata***B***H.zebrina***C, D** sect. Griggsia: **C***H.gigantea***D***H.griggsiana***E–H** sect. Longae: **E***H.curtispatha***F***H.longa***G***H.mariae***H***H.atratensis***I, J** sect. Episcopales: **I***H.episcopalis***J***H.rostrata***K, L** sect. Pastazae: **K***H.pastazae***L***H.rigida*.

The new classification of *Heliconia* includes 17 sections in five subgenera with all clades supported by significant LPP support values > 0.90 (Table 1; Figs 1, 2; Suppl. material 2: fig. S1). Four subgenera (Figs 3, 4) are each composed of two sections (subgen. Heliconiopsis [sects. *Heliconiopsis*, *Perplexae*]; subgen. Stenochlamys [sects. *Stenochlaymys*, *Angustae*], subgen. Taeniostrobus [sects. *Taeniostrobus*, *Aurantiacae*], and subgen. Colubrosae [sects. *Colubrosae*, *Curvae*]). The fifth, subgen. Heliconia (Figs 4, 5), includes nine sections. Four of these sections were previously considered to form distinctive taxonomic subdivisions and are here unequivocally supported as monophyletic entities (sects. *Heliconia*, *Lasia*, *Cannastrum*, and *Longiflorae* [earlier *Zingiberastrum*]). The five remaining sections include species previously scattered and classified among various informal groups and unpublished subdivisions. Here these taxa are sorted into distinct lineages with formal designations (sects. *Lanea*, *Longae*, *Griggsia*, *Episcopales*, and *Pastazae*). Each of the nine sections in subgen. Heliconia has high LPP support (0.92–1.0) and is characterized by a suite of vegetative and reproductive traits. Former subgeneric and sectional names are retained where possible.

**Table 1.** Synopsis of the new classification of *Heliconia* (also see Fig. 2).

**1. *Heliconia* L., Mant. Pl. 2: 147, 211. 1771, nom. cons.**
*Bihai* Miller, Gard. Dict. ed. 4, 1: B1. 1754, nom. rej. (≡ *Heliconia* L.).
**Type.***Heliconiabihai* (L.) L.
**1.1. HeliconiasubgenusHeliconiopsis (Miq.) W.J.Kress, Allertonia 6: 15. 1990.**
*Heliconiopsis* Miq., Fl. Nederl. Ind. 3: 590. 1859.
**Type.***Heliconiopsisamboinensis* Miq. nom. illeg. (≡ *Heliconiabuccinata* Roxb.).
**1.1.1. HeliconiasectionHeliconiopsis (Miq.) W.J.Kress, comb. et stat. nov.**
*Heliconiopsis* Miq., Fl. Nederl. Ind. 3: 590. 1859.
**Type.***Heliconiopsisamboinensis* Miq. (≡ *Heliconiabuccinata* Roxb.).
**1.1.2. HeliconiasectionPerplexae W.J.Kress, sect. nov.**
**Type.***Heliconiaimpudica* Abalo & Morales.
**1.2. HeliconiasubgenusStenochlamys Baker, Ann. Bot. 7: 190, 194–200. 1893.**
**Type.***Heliconiapsittacorum* L. f. (designated by L. Andersson, Opera Bot. 82: 22. 1985).
**1.2.1. HeliconiasectionStenochlamys (Baker) K. Schum., Engler A, ed., Pflanzenr. IV. 45: 37. 1900.**
Heliconiasubgen.Stenochlamys Baker, Ann. Bot. 7: 190, 194–200. 1893.
HeliconiasectionProximochlamys L. Anderss., Opera Bot. 82: 73. 1985. Type: *Heliconiadensiflora* Verlot.
HeliconiasectionZingiberastrum L. Anderss., Opera Bot. 82: 100. 1985. Type: *Heliconiahirsuta* L. f.
**Type.***Heliconiapsittacorum* L. f. (designated by L. Andersson, Opera Bot. 82: 22. 1985).
**1.2.2. HeliconiasectionAngustae W.J.Kress, sect. nov.**
**Type.***Heliconiaangusta* Vell.
**1.3. HeliconiasubgenusTaeniostrobus (Kuntze) Griggs, Bull. Torrey Bot. Club 30: 643. 1903.**
BihaisectionTaeniostrobus Kuntze, Revisio Generum Plantarum. Pars I: 684. 1891.
**Type.***Bihaiimbricata* Kuntze (*Heliconiaimbricata* (Kuntze) Baker (designated by L. Andersson, Flora of Ecuador 22: 11. 1985.)
**1.3.1. HeliconiasectionTaeniostrobus Kuntze**
BihaisectionTaeniostrobus Kuntze, Revisio Generum Plantarum. Pars I: 684. 1891.
HeliconiasectionTortex L. Anderss., Flora of Ecuador 22: 19. 1985. Type. *Heliconialatispatha* Benth.
HeliconiasectionTenebria L. Anderss., Opera Bot. 111: 37. 1992: Type. *Heliconiatenebrosa* Macbr.
**Type.***Bihaiimbricata* Kuntze (*Heliconiaimbricata* (Kuntze) Baker) (designated by L. Andersson, Flora of Ecuador 11: 19. 1985.).
**1.3.2. HeliconiasectionAurantiacae sect. nov.**
**Type.***Heliconiaaurantiaca* Ghiesbr. ex Lemaire.
**1.4. HeliconiasubgenusColubrosae W.J.Kress, subgen. nov.**
**Type.***Heliconiadielsiana* Loes.
**1.4.1. HeliconiasectionColubrosae sect. nov.**
**Type.***Heliconiadielsiana* Loes.
**1.4.2. HeliconiasectionCurvae sect. nov.**
**Type.***Heliconiaburleana* Abalo & Morales.
**1.5. HeliconiasubgenusHeliconia**
**1.5.1. HeliconiasectionHeliconia**
**1.5.2. HeliconiasectionLasia L. Anderss., Opera Bot. 82: 78. 1985.**
**Type.***Heliconiavelutina* L. Anderss.
**1.5.3. HeliconiasectionCannastrum L. Anderss., Opera Bot. 82: 86. 1985.**
**Type.***Heliconiametallica* Planch. & Linden ex. Hook.
**1.5.4. HeliconiasectionLongiflorae sect. nov.**
**Type.***Heliconialongiflora* R.R.Smith.
**1.5.5. HeliconiasectionLanea L. Anderss., Opera Bot. 82: 23, 30. 1985.**
**Type.***Heliconialingulata* Ruiz & Pav.
**1.5.6. HeliconiasectionGriggsia (L. Anderss.) W.J.Kress, comb. et stat. nov.**
HeliconiasubgenusGriggsia L. Anderss., Flora of Ecuador 22: 42. 1985.
**Type.***Heliconiagriggsiana* L.B.Smith.
**1.5.7. HeliconiasectionLongae sect. nov.**
**Type.***Heliconialonga* (Griggs) Winkler.
**1.5.8. HeliconiasectionEpiscopales L. Anderss., Opera Bot. 111: 34. 1992.**
**Type.***Heliconiaepiscopalis* Vell.
**1.5.9. HeliconiasectionPastazae sect. nov.**
**Type.***Heliconiapastazae* L. Anderss.

Provided below for each of the five subgenera and 17 sections are brief descriptions, taxonomic notes, designated types, and included species. “*” indicates placement based on genomic data and morphological traits (136 species); “+” indicates placement based on morphological traits only (51 species); “†” indicates conflict in placement between genomic and morphological data in which the latter evidence was given priority (six species).

#### ﻿1. Heliconia

Taxon classificationPlantaeZingiberalesHeliconiaceae

L., Mant. Pl. 2: 147, 211. 1771 nom. cons.

D1A740C6-264A-51F8-AF45-2FD79ECD17DA

Figs 3 , 4 , 5

Bihai Miller, Gard. Dict. ed. 4, 1: B1. 1754, nom. rej. (≡ Heliconia L.).

##### Type.

*Heliconiabihai* (L.) L.

#### ﻿1.1. Heliconia subgenus Heliconiopsis

Taxon classificationPlantaeZingiberalesHeliconiaceae

(Miq.) W.J.Kress, Allertonia 6: 15. 1990.

C7FF4F31-2515-5488-A885-D2538503613F

Figs 3A–D

Heliconiopsis Miq., Fl. Nederl. Ind. 3: 590. 1859.

##### Type.

*Heliconiopsisamboinensis* Miq. nom. illeg. (≡ *Heliconiabuccinata* Roxb.).

##### Description and taxonomic notes.

Medium- to large-sized rhizomatous herbs with *Musa*-like habit. Inflorescence erect or pendent, with peduncle, rachis and cincinnal bracts green to variously colored with red and yellow; cincinnal bracts distichous or spirally arranged, horizontal to deflexed. Flowers with diurnal or nocturnal anthesis, resupinate and held at right angles to bracts or not resupinate and fully or partially enclosed in bracts; perianth uniformly curved, essentially green or white to green to yellow, variously shaped; ovary green to yellow to orange. Fruits bright red, orange, or blue, glabrous to tomentose. subgenus Heliconiopsis originally included only the six species native to the Asian-Pacific tropics (defined here in sect. Heliconiopsis), but now encompasses at least four additional species from South America contained in sect. Perplexae. Although the latter species are quite distinctive from the paleotropical taxa in inflorescence and flower morphology and color, the phylogenomic analysis provides strong support (LPP = 1.0) for the inclusion of these disparate species in a single subgenus, which is sister to all other heliconias. The morphological characters that link these two sections are not obvious.

##### Distribution.

Tropical Asia-Pacific and Andean South America (Colombia, Ecuador, Fiji, Indonesia, New Caledonia, Papua New Guinea, Samoa, Solomon Islands, Vanuatu).

#### ﻿1.1.1. Heliconia section Heliconiopsis

Taxon classificationPlantaeZingiberalesHeliconiaceae

(Miq.) W.J.Kress, comb. et stat. nov.

88D96CC0-8A37-5CE0-9F1E-3DDED48FB80F

urn:lsid:ipni.org:names:77355024-1

Figs 3A, B

Heliconiopsis Miq., Fl. Nederl. Ind. 3: 590. 1859.

##### Type.

*Heliconiopsisamboinensis* Miq. (≡ *Heliconiabuccinata* Roxb.).

##### Description and taxonomic notes.

Medium- to large-sized rhizomatous herbs with *Musa*-like habit. Inflorescence erect or pendent, with peduncle, rachis and cincinnal bracts green-colored; cincinnal bracts distichous or spirally arranged. Flowers with diurnal or nocturnal anthesis, not resupinate and fully or partially enclosed in bracts; perianth uniformly curved, essentially green; ovary green to yellow to orange. Fruits bright red or orange, glabrous to tomentose. This section includes all six known species native to the Asian-Pacific tropics from Samoa in the South Pacific to New Caledonia. The sectional clade has 1.0 LPP support in the phylogenomic analysis.

##### Species.

*Heliconiaindica Lam. (syn.: *H.buccinata* Roxb.); *H.lanata (Green) W.J.Kress; *H.laufao W.J.Kress; *H.paka A.C.Smith; +*H.papuana* W.J.Kress; *H.solomonensis W.J.Kress.

##### Distribution.

Tropical Asia-Pacific (Fiji, Indonesia, New Caledonia, Papua New Guinea, Samoa, Solomon Islands, Vanuatu).

#### ﻿1.1.2. Heliconia section Perplexae W.J.Kress, sect. nov.

Taxon classificationPlantaeZingiberalesHeliconiaceae

B3A25DC9-BFE4-54ED-87BE-1DC271CD5B4C

urn:lsid:ipni.org:names:77355025-1

Figs 3C, D

##### Type.

*Heliconiaimpudica* Abalo & Morales.

##### Description and taxonomic notes.

Medium-sized rhizomatous herbs with *Musa*-like habit. Inflorescence erect, with peduncle, rachis and cincinnal bracts variously colored with red and yellow; cincinnal bracts distichous or spirally arranged, horizontal to deflexed. Flowers with diurnal anthesis, resupinate; perianth uniformly curved, white to green to yellow, variously shaped; ovary green to yellow. Fruits blue, glabrous. This section includes four species native to Colombia and Ecuador, which were included in subgen. Stenochlamyssect.Lanea by earlier authors (e.g., Andersson 1992; Kress et al. 1999). According to the genomic data, a fifth species, *H.brenneri* Abalo & Morales, is also placed in this section. However, in this classification it is placed in subgen. Heliconiasect.Longae where it shares morphological traits with *H.foreroi* Abalo & Morales and *H.attratensis* Abalo & Morales with erect, red, distichous cincinnal bracts and non-resupinate flowers. Section Perplexae has 1.00 LPP support as a monophyletic group and 1.0 LPP support as sister to sect. Heliconiopsis in the phylogenomic analysis.

##### Species.

*Heliconiagaiboriana Abalo & Morales; *H.impudica Abalo & Morales; *H.virginalis Abalo & Morales; *H.willisiana Abalo & Morales.

##### Distribution.

Tropical Andean South America (Colombia, Ecuador).

#### ﻿1.2. Heliconia subgenus Stenochlamys

Taxon classificationPlantaeZingiberalesHeliconiaceae

Baker, Ann. Bot. 7: 190, 194–200. 1893.

792D9F87-A184-5B55-A254-80F0B7C1286D

Figs 3E–H

##### Type.

*Heliconiapsittacorum* L. f. (designated by L. Andersson, Opera Bot. 82: 22. 1985).

##### Description and taxonomic notes.

Small- to medium-sized rhizomatous herbs with *Musa*-like (rarely *Zingiber*-like) habit. Inflorescence erect, with peduncle, rachis and cincinnal bracts of various colors from red to orange to yellow; cincinnal bracts distichous, generally long and tapering. Flowers with diurnal anthesis, partially to fully resupinate and held at right angles to bracts; perianth short-tubed, angular in cross-section, generally straight to slightly curved, free sepal generally only slightly curved, white to green to yellow, sometimes with green tips; ovary green to yellow to orange to red. Fruits blue, glabrous. The original Heliconiasubgen.Stenochlamys of Baker included 17 species (note that some of these species are not recognized today). Andersson’s (1985a) subgen. Stenochlamys was much expanded into a large and complex taxonomic entity with six sections. In the present classification only 15 species are recognized in two sections (sects. *Stenochlamys* and *Angustae*) of the subgenus. Most of the remaining species are now found in subgen. Heliconia (sects. *Lasia*, *Cannastrum*, and *Longiflorae*). The monophyly of subgen. Stenochlamys has 0.96 LPP support in the molecular analysis and is sister to the remaining three subgenera of *Heliconia*.

##### Distribution.

Tropical Central and South America (Bolivia, Brazil, Colombia, Ecuador, French Guiana, Guyana, Honduras, Nicaragua, Panama, Paraguay, Peru, Suriname, Trinidad & Tobago, Venezuela).

#### ﻿1.2.1. Heliconia section Stenochlamys

Taxon classificationPlantaeZingiberalesHeliconiaceae

(Baker) K. Schum., Engler A, ed., Pflanzenr. IV. 45: 37. 1900.

A555FFB3-405D-5B89-938E-EC88C26B3564

Figs 3E, F

Heliconia subgen. Stenochlamys Baker, Ann. Bot. 7: 190, 194–200. 1893.
Heliconia section Proximochlamys L. Anderss., Opera Bot. 82: 73. 1985. Type: Heliconiadensiflora Verlot.
Heliconia section Zingiberastrum L. Anderss., Opera Bot. 82: 100. 1985. Type: Heliconiahirsuta L. f.

##### Type.

*Heliconiapsittacorum* L. f. (designated by L. Andersson, Opera Bot. 82: 22. 1985).

##### Description and taxonomic notes.

Small- to medium-sized rhizomatous herbs with *Musa*-like (rarely *Zingiber*-like) habit. Inflorescence erect, with peduncle, rachis and cincinnal bracts of various colors from red to orange to yellow; cincinnal bracts distichous, generally long and tapering, usually held erect. Flowers with diurnal anthesis, fully resupinate and held at right angles to bracts; perianth short-tubed, angular in cross-section, generally straight, free sepal generally only slightly curved, yellow to orange (sometimes green or white) with green tips; ovary green to yellow to orange to red. Fruits blue, glabrous. Six of the nine species currently placed in this section were included in Andersson’s sect. Stenochlamys (*H.hirsuta* L. f., *H.densiflora* Verlot and *H.sylvestris* (Gleason) L.B.Smith were placed elsewhere). In the current analysis *H.hirsuta*, despite its position in the molecular analysis as sister to sect. Heliconia, is placed in this section based on the resupinate flowers with green-colored tips and distichous cincinnal bracts. Similarly genomic data placed *H.lourteigiae* Emygdio & Santos in sect. Stenochlamys, but morphological traits more strongly ally this species with others in sect. Taeniostrobus. A LPP support of 0.99 demarcates sect. Stenochlamys in the present classification.

##### Species.

*Heliconiaacuminata L.C.Rich.; *H.brachyantha L. Anderss.; *H.densiflora Verlot; *H.hirsuta L. f. †; *H.psittacorum L. f.; *H.richardiana Miq.; *H.sylvestris (Gleason) L.B.Smith; *H.tarumaensis Barr.; +*H.timothei* L. Anderss.

##### Distribution.

Tropical Central and South America (Bolivia, Brazil, Colombia, Ecuador, French Guiana, Guyana, Honduras, Nicaragua, Panama, Paraguay, Peru, Suriname, Trinidad & Tobago, Venezuela).

#### ﻿1.2.2. Heliconia section Angustae W.J.Kress, sect. nov.

Taxon classificationPlantaeZingiberalesHeliconiaceae

DC86BD04-CDE2-5C9F-B4DB-8585DCF235B1

urn:lsid:ipni.org:names:77355026-1

Figs 3G, H

##### Type.

*Heliconiaangusta* Vell.

##### Description and taxonomic notes.

Small to medium-sized rhizomatous herbs with *Musa*-like habit. Inflorescence erect, with peduncle, rachis and cincinnal bracts colored primarily red; cincinnal bracts distichous usually held erect at 60–90 degrees to horizontal. Flowers with diurnal anthesis, partially resupinate; perianth straight to uniformly curved, white to green to yellow, triangular in cross-section; ovary yellow to red. Fruits blue, glabrous. The six species united here in subgen. Stenochlamyssect.Angustae were formerly distributed across three sections by [Bibr B5][Bibr B7]; sects. *Stenochalmys*, *Tortex* [now *Taeniostrobus*], and *Lasia*). Section Angustae with 1.0 LPP support as a monophyletic group in the molecular analysis is sister to sect. Stenochlamys.

##### Species.

*Heliconiaangusta Vell.; +*H.dasyantha* Koch & Bouché; *H.farinosa Raddi; *H.laneana Barreiros; *H.sampaioana L. Emygdio; *H.velloziana L. Emygdio.

##### Distribution.

Tropical Northern South America (Brazil, French Guiana, Suriname).

#### ﻿1.3. Heliconia subgenus Taeniostrobus

Taxon classificationPlantaeZingiberalesHeliconiaceae

(Kuntze) Griggs, Bull. Torrey Bot. Club 30: 643. 1903.

07D085CE-853A-5393-821F-BC05C2E06A10

Figs 3I–L

Bihai section Taeniostrobus Kuntze, Revisio Generum Plantarum. Pars I: 684. 1891.

##### Type.

*Bihaiimbricata* Kuntze (*Heliconiaimbricata* (Kuntze) Baker (designated by L. Andersson, Flora of Ecuador 22: 11. 1985.).

##### Description and taxonomic notes.

Small- to large-sized rhizomatous herbs with *Musa*-, *Canna*-, or *Zingiber*-like habit. Inflorescence erect (rarely pendent), with peduncle, rachis and cincinnal bracts of various colors from pink to red to orange to yellow; cincinnal bracts distichous or spirally arranged. Flowers with diurnal anthesis, not resupinate and fully or partially enclosed in bracts or fully resupinate and held at right angles to bracts; perianth uniformly curved (rarely s-shaped), white to green to yellow; ovary green to yellow to red. Fruits blue, glabrous. subgenus Taeniostrobus in the current classification is made up of two sections (sects. *Taeniostrobus* and *Aurantiacae*) and includes species from four earlier designated subgenera (subgens. *Taeniostrobus*, *Heliconia*, *Stenochlamys*, and *Griggsia*), although the majority of species were included in Andersson’s subgen. Heliconiasect.Tortex. Few, if any, morphological traits are shared by all species across subgen. Taeniostrobus. The very high strong support (LPP = 1.0) in the phylogenomic analysis is primary evidence for uniting these species into a single monophyletic subgenus with two somewhat distinct sections.

##### Distribution.

Tropical Mexico, Central, and South America (Belize, Brazil, Colombia, Costa Rica, Ecuador, El Salvador, French Guiana, Guatemala, Honduras, Mexico, Nicaragua, Panama, Peru, Suriname, Venezuela).

#### ﻿1.3.1. Heliconia section Taeniostrobus

Taxon classificationPlantaeZingiberalesHeliconiaceae

Kuntze

A4498207-A255-5221-8DB7-DCF0E223414F

Figs 3I, J

Bihai section Taeniostrobus Kuntze, Revisio Generum Plantarum. Pars I: 684. 1891.
Heliconia section Tortex L. Anderss., Flora of Ecuador 22: 19. 1985. Type. Heliconialatispatha Benth.
Heliconia section Tenebria L. Anderss., Opera Bot. 111: 37. 1992: Type. Heliconiatenebrosa Macbr.

##### Type.

*Bihaiimbricata* Kuntze (*Heliconiaimbricata* (Kuntze) Baker) (designated by L. Andersson, Flora of Ecuador 11: 19. 1985.).

##### Description and taxonomic notes.

Medium- to large-sized rhizomatous herbs with *Musa*-like habit. Inflorescence erect (rarely pendent), with peduncle, rachis and cincinnal bracts of various colors from pink to red to orange to yellow; cincinnal bracts distichous or spirally arranged. Flowers with diurnal anthesis, not resupinate (or rarely so), and fully or partially enclosed in bracts; perianth uniformly curved (rarely s-shaped), white to green to yellow; ovary green to yellow to red. Fruits blue, glabrous. Species in this section can be quite variable in most reproductive features, although almost all have non-resupinate flowers enclosed in the cincinnal bracts. The 0.99 LPP support identifies this clade as distinct from its sister taxon sect. Aurantiacae. The genomic data place *H.lourteigiae* L. Emygdio & Santos in sect. Stenochlamys, however, the shape and orientation of the cincinnal bracts and non-resupinate, enclosed flowers suggest that this species is more properly placed in sect. Taeniostrobus.

##### Species.

*Heliconiaatropurpurea Daniels & Stiles; *H.barryana W.J.Kress; *H.beckneri R.R. Smith; *H.bella W.J.Kress; *H.bourgaeana O.G. Peters.; *H.champneiana Griggs; *H.clinophila R.R.Smith; *H.collinsiana Griggs; +*H.cucullata* W.J.Kress & L.Anderss.; +*H.darienensis* L.Anderss.; *H.faunorum W.J.Kress & L.Anderss.; *H.gracilis Daniels & Stiles; *H.ignescens Daniels & Stiles; *H.imbricata (Kuntze) Baker; *H.irrasa R.R.Smith; *H.lankesteri Standl.; *H.latispatha Benth.; *H.librata Griggs; *H.lindsayana W.J.Kress; *H.lophocarpa Daniels & Stiles; *H.lourteigiae L.Emygdio & Santos †; *H.lutea W.J.Kress; +*H.monteverdensis* Daniels & Stiles; +*H.mooreana* R.R.Smith; *H.nutans Woodson; *H.reticulata (Griggs) Winkl.; +*H.rodriguezii* Stiles; +*H.sarapiquensis* Daniels & Stiles; *H.secunda Daniels & Stiles; *H.spathocircinata Aristeg.; +*H.tenebrosa* Macbr.; *H.thomasiana W.J.Kress; *H.tortuosa Griggs; *H.umbrophila Daniels & Stiles.

##### Distribution.

Tropical Mexico, Central, and South America (Belize, Brazil, Colombia, Costa Rica, Ecuador, El Salvador, French Guiana, Guatemala, Honduras, Mexico, Nicaragua, Panama, Peru, Suriname, Venezuela).

#### ﻿1.3.2. Heliconia section Aurantiacae W.J.Kress, sect. nov.

Taxon classificationPlantaeZingiberalesHeliconiaceae

D4D9A0BC-C77F-50C2-A446-4B00A09A8628

urn:lsid:ipni.org:names:77355027-1

Figs 3K, L

##### Type.

*Heliconiaaurantiaca* Ghiesbr. ex Lemaire.

##### Description and taxonomic notes.

Small- to medium-sized rhizomatous herbs with *Musa*- to *Zingiber*-like habit. Inflorescence erect, with peduncle, rachis and cincinnal bracts red to orange; cincinnal bracts spirally arranged (rarely distichous). Flowers with diurnal anthesis, fully resupinate and held at right angles to bracts; perianth uniformly curved, green to yellow; ovary green to yellow. Fruits blue, glabrous. The five species united here in subgen. Taeniostrobussect.Aurantiacae were formerly placed in two sections by Andersson (1992; sects. *Lanea* and *Zingiberastrum* [now *Longiflorae*]). The species in this section, with primarily spirally arranged bracts, resupinate flowers, and some with *Zingiber*-like leaf arrangement, are distinct from taxa in sect. Taeniostrobus and are supported in the molecular analysis with 0.92 LPP support as a monophyletic group sister to that section.

##### Species.

*Heliconiaadflexa (Griggs) Standl.; *H.aurantiaca Ghiesbr. ex Lemaire; *H.crassa Griggs; *H.schiedeana Kl.; *H.spissa Griggs.

##### Distribution.

Tropical Mexico and Central America (Belize, Costa Rica, Guatemala, Honduras, Mexico, Nicaragua).

#### ﻿1.4. Heliconia subgenus Colubrosae W.J.Kress, subgen. nov.

Taxon classificationPlantaeZingiberalesHeliconiaceae

EAA95F15-7F12-54A0-BDF6-19DB5AE144A1

urn:lsid:ipni.org:names:77355028-1

Figs 4A–D

##### Type.

*Heliconiadielsiana* Loes.

##### Description and taxonomic notes.

Medium- to large-sized rhizomatous herbs with *Musa*-like habit. Inflorescence pendent or erect, with peduncle, rachis, and cincinnal bracts usually entirely red, sometimes green distally (rarely yellow); cincinnal bracts distichous or spirally arranged, horizontal or reflexed, usually elongate and tapering. Flowers with diurnal anthesis, fully or partially enclosed in bracts or resupinate and held at right angles to bracts; perianth s-shaped (sigmoid) or c-shaped (sharply curved), free sepal extended or not and reflexed, fused sepals reflexed at tips or not, generally yellow, sometimes white to green; ovary yellow to white (rarely green). Fruits blue, glabrous. The numerous species placed here in subgen. Colubrosae were formerly included in either subgen. Griggsia (with pendent inflorescences) or subgen. Stenochlamys (with erect inflorescences) by both Andersson (1985a, 1992) and Kress et al. (1999), but these two subgenera were never closely associated. The 1.0 LPP support in the molecular analysis strongly unites the species in these two subgenera into a single monophyletic lineage.

##### Distribution.

Tropical Central and South America (Bolivia, Brazil, Colombia, Costa Rica, Ecuador, French Guiana, Guyana, Panama, Peru, Venezuela).

#### ﻿1.4.1. Heliconia section Colubrosae W.J.Kress, sect. nov.

Taxon classificationPlantaeZingiberalesHeliconiaceae

51CEDC03-5DA4-59C5-87C2-88C55EBCEDD2

urn:lsid:ipni.org:names:77355029-1

Figs 4A, B

##### Type.

*Heliconiadielsiana* Loes.

##### Description and taxonomic notes.

Medium- to large-sized rhizomatous herbs with *Musa*-like habit. Inflorescence pendent, with peduncle, rachis, and cincinnal bracts usually entirely red, sometimes green distally (rarely yellow); cincinnal bracts distichous or spirally arranged, horizontal or reflexed, usually elongate and tapering. Flowers with diurnal anthesis, fully or partially enclosed in bracts; perianth s-shaped (sigmoid), free sepal extended and reflexed, fused sepals reflexed at tips, generally yellow, sometimes white to green; ovary yellow to white. Fruits blue, glabrous. The species placed in sect. Colubrosae were formerly included in subgen. Griggsia by both Andersson (1992) and Kress et al. (1999) with the majority of species placed in either the informal *H.trichocarpa* group or *H.obscura* group by Andersson (1992) and the unpublished sects. *Sigmoideae*, *Pendulae*, *Obscurae*, and *Dromadarius* by Kress et al. (1999). Section Colubrosae is the largest group of species in the genus with pendent inflorescences and the 1.0 LPP support in the molecular analysis strongly unites monophyletic sect. Colubrosae sister to sect. Curvae. The genomic data place *H.pendula* Wawra in sect. Heliconia, however, the pendent inflorescence with spirally arranged bracts, extended and reflexed free sepals, and fused sepals reflexed at the tips suggest that this species is more properly placed in sect. Colubrosae.

##### Species.

+*Heliconiabadilloi* Abalo & Morales; +*H.berriziana* Abalo & Morales; +*H.caquetensis* Abalo & Morales; +*H.chrysocraspeda* Abalo & Morales; *H.colgantea R.R.Smith ex Daniels & Stiles; +*H.combinata* Abalo & Morales; *H.dielsiana Loes.; +*H.estiletioides* Abalo & Morales; +*H.fernandezii* Abalo & Morales; +*H.fredberryana* W.J.Kress; *H.huilensis Abalo & Morales; +*H.intermedia* Abalo & Morales; +*H.laxa* Abalo & Morales; +*H.lentiginosa* Abalo & Morales; *H.lozanoi Abalo & Morales; *H.maculata W.J.Kress; +*H.mucilagina* Abalo & Morales; *H.mutisiana Cuatrecasas; +*H.nariniensis* Abalo & Morales; *H.necrobracteata W.J.Kress; +*H.nitida* Abalo & Morales; *H.obscura Abalo & Morales; *H.obscuroides L. Anderss.; *H.oleosa Abalo & Morales; *H.pendula Wawra †; +*H.peteriana* Abalo & Morales; *H.reptans Abalo & Morales; *H.riopalenquensis Dodson & A. Gentry; +*H.robertoi* Abalo & Morales; *H.robusta Pax; *H.sclerotricha Abalo & Morales; +*H.signa-hispanica* Abalo & Morales; *H.talamancana Daniels & Stiles; *H.trichocarpa Daniels & Stiles; +*H.villosa* Kl.

##### Distribution.

Tropical Central and South America (Bolivia, Brazil, Colombia, Costa Rica, Ecuador, French Guiana, Guyana, Panama, Peru, Venezuela).

#### ﻿1.4.2. Heliconia section Curvae W.J.Kress, sect. nov.

Taxon classificationPlantaeZingiberalesHeliconiaceae

FA34BC6D-0DEF-572A-AE7D-4C814B1D3999

urn:lsid:ipni.org:names:77355030-1

Figs 4C, D

##### Type.

*Heliconiaburleana* Abalo & Morales.

##### Description and taxonomic notes.

Medium- to large-sized rhizomatous herbs with *Musa*-like habit. Inflorescence erect with peduncle, rachis, and cincinnal bracts entirely red or yellow; cincinnal bracts distichous or spirally arranged, generally horizontal, elongate and tapering. Flowers with diurnal anthesis, resupinate and held at right angles to bracts; perianth c-shaped (sharply curved), free sepal extended and reflexed, fused sepals not reflexed at tips, yellow, or white to green; ovary yellow or green. Fruits blue, glabrous. For the most part the species placed here in sect. Curvae were formerly included in subgen. Stenochlamyssect.Lanea by both Andersson (1985a, 1992) and Kress et al. (1999). One species (*H.sanctae-martae* L. Anderss.) was included in sect. Cannastrum by Kress et al. (1999). The 1.0 LPP support in the molecular analysis together with the floral features listed here unite these four species into a single monophyletic lineage at the sectional level sister to sect. Colubrosae.

##### Species.

*Heliconiaaristeguietae Abalo & Morales; *H.burleana Abalo & Morales; *H.gilbertiana Abalo & Morales; +*H.sanctae-martae* L. Anderss.

##### Distribution.

Primarily Tropical Andean South America (Colombia, Ecuador, Panama, Peru).

#### ﻿1.5. Heliconia subgenus Heliconia

Taxon classificationPlantaeZingiberalesHeliconiaceae

36BEF6CF-D029-5037-A25C-C650B685D10A

Figs 4E–L , 5A–L

##### Description and taxonomic notes.

Medium- to large-sized rhizomatous herbs with *Musa*-, *Canna*-, or *Zingiber*-like habit. Inflorescence erect or pendent, with peduncle, rachis and cincinnal bracts of various colors from red to orange to yellow to green; cincinnal bracts distichous or spirally arranged, congested or widely separated. Flowers with diurnal anthesis, not resupinate and fully or partially enclosed in bracts or fully resupinate and held at right angles to bracts; perianth with short to elongate tube, straight to uniformly curved to s-shaped (sigmoid), white to green to yellow to pink to red, glabrous to sometimes hairy; ovary white to green to yellow to red, generally glabrous. Fruits blue, glabrous (rarely hirsute). The size and complexity of subgen. Heliconia has varied with author, including from four (Andersson 1992) to six (Kress et al. 1999) sections. The molecular data provide 0.9 LPP support for a newly defined subgen. Heliconia, which includes many taxa formerly placed in subgenera *Stenochlamys* and *Griggsia*, e.g., from sects. *Lasia*, *Cannastrum*, *Zingiberastrum* (now *Longiflorae*), and parts of *Lanea*, as well as the informal species groups and unpublished sects. *Griggsia*, *Barbatae*, *Arcuatae*, *Longae*, *Rostratae*, *Pendulae*, and *Retiformes*. The wide variance in morphological traits characterizing species in subgen. Heliconia in part accounts for this complex arrangement of taxa. In the current classification nine sections, each supported by 0.92–1.0 LPP, are recognized. Four sections comprise an unresolved polyphyletic assemblage within the subgenus while the other five sections form a monophyletic group with 1.0 LPP support.

##### Distribution.

Tropical Mexico, Central America, South America, and the Caribbean (Argentina, Barbados, Belize, Bolivia, Brazil, Colombia, Costa Rica, Dominica, Dominican Republic, Ecuador, El Salvador, French Guiana, Grenada, Guadeloupe, Guatemala, Guyana, Haiti, Honduras, Jamaica, Martinique, Mexico, Montserrat, Nicaragua, Panama, Paraguay, Peru, Puerto Rico, Saba, St. Eustatius, St. Kitts & Nevis, St. Lucia, St. Vincent & the Grenadines, Suriname, Trinidad & Tobago, Venezuela).

#### ﻿1.5.1. Heliconia section Heliconia

Taxon classificationPlantaeZingiberalesHeliconiaceae

F79A7CB3-00DB-55DC-B2DA-4C44CBE047F7

Figs 4E, F

##### Description and taxonomic notes.

Generally large-sized rhizomatous herbs with *Musa*-like habit. Inflorescence erect, with peduncle, rachis and cincinnal bracts of various colors from red to orange to yellow to green, margins often green; cincinnal bracts distichous, congested and usually overlapping. Flowers with diurnal anthesis, not resupinate and fully enclosed in bracts; perianth generally with elongate tube, c-shaped (sharply curved) to s-shaped (sigmoid), hidden within bract with only apex protruding, white with green apex, glabrous; ovary white, glabrous. Fruits blue, glabrous, protruding from bract on elongated pedicel at maturity. Two species were placed in the genomic analyses as basal taxa in the clade including sect. Heliconia. However, morphological features indicate that *H.hirsuta* L. f. is more properly including in sect. Stenochlamys and *H.pendula* Wawra in sect Colubrosae. The close relationship among the species in the current sect. Heliconia has long been recognized by all authors. The 1.0 LPP support for this monophyletic group in the molecular analysis confirms this observation.

##### Species.

*Heliconiaaurea Rodríguez; *H.bihai (L.) L.; *H.caribaea Lam.; *H.lennartiana W.J.Kress; *H.orthotricha L. Anderss.; *H.rodriguensis Aristeg.; *H.stricta Huber; *H.wagneriana O.G.Peters.

##### Distribution.

Tropical Mexico, Central America, South America, and the Caribbean (Barbados, Belize, Bolivia, Brazil, Colombia, Costa Rica, Dominica, Dominican Republic, Ecuador, French Guiana, Grenada, Guadeloupe, Guatemala, Guyana, Haiti, Honduras, Jamaica, Martinique, Mexico, Montserrat, Nicaragua, Panama, Peru, Puerto Rico, Saba, St. Eustatius, St. Kitts & Nevis, St. Lucia, St. Vincent & the Grenadines, Suriname, Trinidad & Tobago, Venezuela).

#### ﻿1.5.2. Heliconia section Lasia

Taxon classificationPlantaeZingiberalesHeliconiaceae

L. Anderss., Opera Bot. 82: 78. 1985.

E3C5F8B6-12BC-5AAC-8C76-39712E7C6CF0

Figs 4G, H

##### Type.

*Heliconiavelutina* L. Anderss.

##### Description and taxonomic notes.

Medium-sized rhizomatous herbs with *Musa*-like habit. Inflorescence erect, with peduncle and rachis usually yellow, cincinnal bracts red, often yellow near rachis, usually hirsute; cincinnal bracts distichous, widely spaced, elongate and tapering. Flowers with diurnal anthesis, fully exposed at maturity, resupinate and held at nearly right angles to bracts; perianth with short tube, straight to uniformly curved, yellow to green (rarely red), glabrous; ovary green to yellow, glabrous. Fruits blue, glabrous (rarely hirsute). sect. Lasia was placed in subgen. Stenochlamys originally by Andersson (1985a, 1992) and followed by Kress et al. (1999). In the present classification molecular data support its inclusion in subgen. Heliconia although few morphological traits support that placement and only two of the four species in the section were included in the analysis. The 1.0 LPP support clearly unites these two species in a monophyletic section, but its relationship to other sections in the subgenus is equivocal at this time.

##### Species.

+*Heliconiaestherae* Abalo & Morales; *H.julianii Barreiros; *H.lasiorachis L. Anderss.; +*H.velutina* L. Anderss.

##### Distribution.

Tropical South America (Bolivia, Brazil, Colombia, Ecuador, Peru, Venezuela).

#### ﻿1.5.3. Heliconia section Cannastrum

Taxon classificationPlantaeZingiberalesHeliconiaceae

L. Anderss., Opera Bot. 82: 86. 1985.

8C666321-3C6F-5BAF-B6CF-2865BC42B3B2

Figs 4I, J

##### Type.

*Heliconiametallica* Planch. & Linden ex. Hook.

##### Description and taxonomic notes.

Medium- to large-sized rhizomatous herbs with *Canna*- to rarely *Zingiber*-like habit. Inflorescence erect, with peduncle, rachis and cincinnal bracts usually red (rarely yellow to green); cincinnal bracts distichous, generally separated. Flowers with diurnal anthesis, fully exposed at maturity, resupinate and held at right angles to bracts; perianth with short to medium-sized tube, straight to uniformly curved, yellow (rarely pink), sometimes with green towards apex, glabrous; ovary green to yellow to red, sometimes with green apex, glabrous. Fruits blue, glabrous. Section Cannastrum was placed in subgen. Stenochlamys originally by Andersson (1985a, 1992) and followed by Kress et al. (1999). In the present classification molecular data support its inclusion in subgen. Heliconia although few morphological traits support that placement. The 1.0 LPP support clearly unites the species in this section as a monophyletic group. The genomic data place one species, *H.pardoi* Abalo & Morales, in sect. Griggsia, but the vegetative, inflorescence and floral traits suggest its inclusion in sect. Cannastrum. The relationship of this section to others in the subgenus is equivocal at this time.

##### Species.

+*Heliconiaberryi* Abalo & Morales; *H.calatheaphylla Daniels & Stiles; *H.deflexa Daniels & Stiles; *H.golfodulcensis Daniels & Stiles; *H.mathiasiae Daniels & Stiles; *H.meridensis Kl.; *H.metallica Planch. & Linden ex. Hook.; +*H.mincana* Abalo & Morales; +*H.montana* Abalo & Morales; *H.osaensis Cuf.; *H.pardoi Abalo & Morales †; *H.vaginalis Benth.; +*H.venusta* Abalo & Morales; *H.wilsonii Daniels & Stiles.

##### Distribution.

Tropical Mexico, Central America, and South America (Belize, Bolivia, Brazil, Colombia, Costa Rica, Ecuador, El Salvador, Guatemala, Honduras, Mexico, Nicaragua, Panama, Peru, Venezuela).

#### ﻿1.5.4. Heliconia section Longiflorae W.J.Kress, sect. nov.

Taxon classificationPlantaeZingiberalesHeliconiaceae

F29C7FCA-1FD4-586A-898B-77714DCDB632

urn:lsid:ipni.org:names:77355031-1

Figs 4K, L

##### Type.

*Heliconialongiflora* R.R.Smith.

##### Description and taxonomic notes.

Small to medium-sized rhizomatous herbs with *Zingiber*-like habit. Inflorescence erect with peduncle and rachis yellow or green and cincinnal bracts red with orange to yellow towards rachis; cincinnal bracts distichous and widely separated. Flowers with diurnal anthesis, fully exposed at maturity, resupinate and held at right angles to bracts; perianth generally straight to variously curved, white to yellow to orange, sometimes green towards apex, glabrous; ovary white to yellow to orange with green apex, glabrous. Fruits blue, glabrous. The *Zingiber*-like habit of the shoots is a shared featured of the species in this section, which has 1.0 LPP support in the molecular analysis. Andersson (1985a) previously named this section “*Zingiberastrum*” and designated *H.hirsuta* as the type. However, *H.hirsuta* L. f. is placed in sect. Stenochlamys in the present classification and the section is renamed sect. Longiflorae with the designation of a new type.

##### Species.

+*Heliconiaapparicioi* Barreiros; *H.cordata L. Anderss.; +*Heliconiaecuadoriensis* (L. Anderss.) W.J.Kress, comb. et stat. nov. (Heliconialongiflorassp.ecuadoriensis L. Anderss., Opera Bot. 82: 113. 1985. Type. Asplund 16457. Ecuador, Esmeraldas, Timbre [San Mateo], 24 May 1955 [S]); *H.longiflora R.R.Smith; *H.schumanniana Loes.; +*H.tacarcunae* L.Anderss.

##### Distribution.

Tropical Central America and South America (Brazil, Colombia, Costa Rica, Ecuador, Nicaragua, Panama, Peru).

#### ﻿1.5.5. Heliconia section Lanea

Taxon classificationPlantaeZingiberalesHeliconiaceae

L. Anderss., Opera Bot. 82: 23, 30. 1985.

0AA535CB-B679-5E7C-82AA-6D41525F9070

Figs 5A, B

##### Type.

*Heliconialingulata* Ruiz & Pav.

##### Description and taxonomic notes.

Medium- to large-sized rhizomatous herbs with *Musa*-like habit. Inflorescence erect, with peduncle, rachis and cincinnal bracts of various colors from pink to red to yellow; cincinnal bracts distichous or spirally arranged, usually widely separated. Flowers with diurnal anthesis, fully exposed at maturity, resupinate and held at right angles to bracts; perianth generally with short to medium-sized tube, straight to uniformly curved, green to yellow, glabrous; ovary green to yellow to red, generally glabrous. Fruits blue, glabrous. Section Lanea includes species which were formerly placed in sects. *Lanea* or *Cannastrum* by Andersson (1985a, 1992). Here these six species comprise a monophyletic group that is strongly supported (LPP = 1.0) as sister to a lineage of species with pendent inflorescences which were formerly classified in subgen. Griggsia by Andersson (1992) and Kress et al. (1999).

##### Species.

*Heliconiaaemygdiana Burle-Marx; +*H.fugax* L. Anderss.; *H.gloriosa Abalo & Morales; *H.lingulata Ruiz & Pav.; *H.subulata Ruiz & Pav.; *H.zebrina Plowman, W.J.Kress & Kennedy.

##### Distribution.

Tropical South America (Argentina, Bolivia, Brazil, Colombia, Ecuador, Paraguay, Peru, Suriname).

#### ﻿1.5.6. Heliconia section Griggsia

Taxon classificationPlantaeZingiberalesHeliconiaceae

(L. Anderss.) W.J.Kress, comb. et stat. nov.

E57EB7F3-E90E-59BF-B31A-B391619007A5

urn:lsid:ipni.org:names:77355032-1

Figs 5C, D

Heliconia subgenus Griggsia L. Anderss., Flora of Ecuador 22: 42. 1985.

##### Type.

*Heliconiagriggsiana* L.B.Smith.

##### Description and taxonomic notes.

Large-sized rhizomatous herbs with *Musa*-like habit. Inflorescence pendent, with peduncle, rachis and cincinnal bracts red to pink to green; cincinnal bracts distichous or sub-spirally arranged, partially separated. Flowers with diurnal anthesis, not resupinate and fully enclosed in bracts; perianth with short to medium-sized tube, uniformly curved, yellow to orange, glabrous; ovary white to red, glabrous. Fruits blue, glabrous. subgenus Griggsia was established by Andersson (1985b, 1992) to include all species of *Heliconia* with pendent inflorescences. Here it is recognized as a section with only three species (LPP = 1.0) and is clearly allied to the other three sections with pendent inflorescences in this subgenus. In the molecular analyses *H.pardoi* Abalo & Morales was indicated as allied to species in sect. Griggsia, but shares morphological features with taxa in sect. Cannastrum, where it is placed in this classification. A second species, *H.titanum* W.J.Kress & J. Betancur, was placed in sect. Longae by the genomic data, which may support an earlier suggestion (C. Black, pers. comm.) that this species is a hybrid with one parental species in that section. Here it is placed in sect. Griggsia.

##### Species.

*Heliconiagigantea W.J.Kress & J. Betancur; *H.griggsiana L.B.Smith; *H.titanum W.J.Kress & J.Betancur †.

##### Distribution.

Tropical Andean South America (Colombia, Ecuador).

#### ﻿1.5.7. Heliconia section Longae W.J.Kress, sect. nov.

Taxon classificationPlantaeZingiberalesHeliconiaceae

4F90AE52-FA5D-50E5-948E-EA4877EE429E

urn:lsid:ipni.org:names:77355033-1

Figs 5E–H

##### Type.

*Heliconialonga* (Griggs) Winkler.

##### Description and taxonomic notes.

Medium- to large-sized rhizomatous herbs with *Musa*-like habit. Inflorescence generally pendent or occasionally erect, with peduncle, rachis and cincinnal bracts almost always red to rarely yellow; cincinnal bracts distichous or slowly spirally arranged, congested or widely separated. Flowers with diurnal anthesis, not resupinate and fully enclosed in bracts; perianth generally with medium-sized tube, uniformly curved to c-shaped to rarely s-shaped, yellow to white to rarely pink or red, glabrous to hairy; ovary white to yellow to red, generally glabrous. Fruits blue, glabrous. Section Longae is the second largest group of species with pendent inflorescences next to sect. Colubrosae. Species in this section have previously been informally included in the *H.pogonantha*, *H.longa*, and *H.grigssiana* groups by Andersson (1992) and scattered among seven unpublished sections (*Arcuatae*, *Barbatae*, *Dromedarius*, *Obscurae*, *Longae*, and *Retiformes*) by Kress et al. (1999). Three species in sect. Longae have erect inflorescences (formerly in the unpublished section Complanatae). One of these species, *H.brenneri* Abalo & Morales, was included in subgen. Heliconiopsissect.Perplexae according to the genomic data, but is morphologically similar to the other two species with erect inflorescences in sect. Longae (*H.foreroi* Abalo & Morales and *H.attratensis* Abalo & Morales) sharing red, distichous cincinnal bracts, and non-resupinate flowers and is placed in this section. Tissue was not available for many of the species with pendent inflorescence in the current phylogenomic analyses and future investigations may shuffle some of these species between sect. Longae and sect. Colubrosae. Section Longae is closely allied by 0.9 LPP support to the other three sections in subgen. Heliconia characterized by pendent inflorescences (sects. *Griggsia*, *Episcopales*, and *Pastazae*).

##### Species.

+*Heliconiaarrecta* W.J.Kress & J. Betancur; *H.atratensis Abalo & Morales; *H.berguidoi R. Flores, C. Black & A. Ibáñez; *H.brenneri Abalo & Morales †; *H.carmelae Abalo & Morales; *H.curtispatha Peters.; *H.danielsiana W.J.Kress; +*H.donstonea* W.J.Kress & J. Betancur; *H.excelsa L. Anderss.; +*H.foreroi* Abalo & Morales; *H.fragilis Abalo & Morales; *H.harlingii L. Anderss.; +*H.holmquistiana* Abalo & Morales; *H.longa (Griggs) Winkler; +*H.lutheri* W.J.Kress; *H.magnifica W.J.Kress; *H.mariae Hook. f.; +*H.markiana* Abalo & Morales; *H.nigripraefixa Dodson & Gentry; +*H.paludigena* Abalo & Morales; *H.pogonantha Cuf.; *H.ramonensis Daniels & Stiles; +*H.regalis* L. Anderss.; *H.rhodantha Abalo & Morales; +*H.samperiana* W.J.Kress & J. Betancur; +*H.sanctae-theresae* Abalo & Morales; *H.spiralis Abalo & Morales; +*H.stella-maris* Abalo & Morales; *H.stilesii W.J.Kress; *H.terciopela W.J.Kress & J. Betancur; *H.vellerigera Poepp.; *H.xanthovillosa W.J.Kress.

##### Distribution.

Tropical Central America and South America (Belize, Colombia, Costa Rica, Ecuador, Guatemala, Honduras, Nicaragua, Panama, Peru, Venezuela).

#### ﻿1.5.8. Heliconia section Episcopales

Taxon classificationPlantaeZingiberalesHeliconiaceae

L. Anderss., Opera Bot. 111: 34. 1992.

2E329AAB-D1A4-557D-A856-79D3871008DB

Figs 5I, J

##### Type.

*Heliconiaepiscopalis* Vell.

##### Description and taxonomic notes.

Medium- to large-sized rhizomatous herbs with *Musa*-like habit. Inflorescence pendent or erect, with peduncle and rachis generally red and cincinnal bracts red to pink with green towards tip; cincinnal bracts distichous or rarely spirally arranged, moderated separated (rarely congested). Flowers with diurnal anthesis, not resupinate and fully enclosed in bracts; perianth with medium-sized tube, uniformly curved, white to yellow, glabrous; ovary white to yellow, glabrous. Fruits blue, glabrous. Nearly all of the species in sect. Episcopales (earlier referred to as “sect. Rostratae ined.” by Kress et al. 1999) have been considered as closely related because of their shared pendent inflorescences and flower features. The placement in this group of *H.episcopalis* Vell. with an erect inflorescence and congested bracts suggests that this inflorescence type has an independent evolutionary origin in this lineage. The closest relatives of *H.penduloides* Loes. have always been puzzling, but the 0.97 LPP support clearly unites these six species into a monophyletic group.

##### Species.

*Heliconiaepiscopalis Vell.; *H.juruana Loes.; *H.marginata (Griggs) Pittier; *H.penduloides Loes; *H.rostrata Ruiz & Pav.; *H.standleyi Macbr.

##### Distribution.

Tropical Central and South America (Bolivia, Brazil, Colombia, Costa Rica, Ecuador, French Guiana, Guyana, Panama, Peru, Trinidad & Tobago, Venezuela).

#### ﻿1.5.9. Heliconia section Pastazae W.J.Kress, sect. nov.

Taxon classificationPlantaeZingiberalesHeliconiaceae

65F73CA6-54B2-56FD-8D9C-656140863F1C

urn:lsid:ipni.org:names:77355034-1

Figs 5K, L

##### Type.

*Heliconiapastazae* L. Anderss.

##### Description and taxonomic notes.

Medium-sized rhizomatous herbs with *Musa*-like habit. Inflorescence pendent, with peduncle, rachis and cincinnal bracts usually red with yellow distally along margins (rarely completely yellow or green); cincinnal bracts sub-distichous to spirally arranged, separated. Flowers with diurnal anthesis, not resupinate and fully to partially enclosed in bracts; perianth with short to medium-sized tube, uniformly curved, yellow to green, glabrous; ovary white to green to yellow, glabrous. Fruits blue, glabrous. Most of the species in this section have been considered as related and informally placed in the *H.platystachys* group (Andersson 1985b, 1992) or the unpublished sect. Pendulae (Kress et al., 1999). The strong LPP support (0.92) for sect. Pastazae is further evidence for its recognition as a distinct monophyletic lineage within subgen. Heliconia.

##### Species.

*Heliconiaabaloi G. Morales; *H.chartacea Lane ex Barreiros; *H.pastazae L. Anderss.; *H.platystachys Baker; +*H.rigida* Abalo & Morales.

##### Distribution.

Tropical Central America and South America (Brazil, Colombia, Costa Rica, Ecuador, French Guiana, Guyana, Panama, Peru, Venezuela).

## ﻿Conclusions

The phylogenomic patterns of species relationships revealed here coupled with the newly proposed classification can now be used to further our understanding of the ecological patterns and interactions of heliconias in nature. Moreover, the results can aid in enhancing the economic potential of these plants in the horticultural trade by identifying prospective hybridization partners as well as species with shared cultivation and ornamental traits for introduction into sustainable commerce. As more genomic data are added to analyses and field observations of morphological features are increased, the current classification of *Heliconia* will undoubtedly be modified and refined as all classifications have undergone in the past.

## Supplementary Material

XML Treatment for Heliconia

XML Treatment for Heliconia subgenus Heliconiopsis

XML Treatment for Heliconia section Heliconiopsis

XML Treatment for Heliconia section Perplexae W.J.Kress, sect. nov.

XML Treatment for Heliconia subgenus Stenochlamys

XML Treatment for Heliconia section Stenochlamys

XML Treatment for Heliconia section Angustae W.J.Kress, sect. nov.

XML Treatment for Heliconia subgenus Taeniostrobus

XML Treatment for Heliconia section Taeniostrobus

XML Treatment for Heliconia section Aurantiacae W.J.Kress, sect. nov.

XML Treatment for Heliconia subgenus Colubrosae W.J.Kress, subgen. nov.

XML Treatment for Heliconia section Colubrosae W.J.Kress, sect. nov.

XML Treatment for Heliconia section Curvae W.J.Kress, sect. nov.

XML Treatment for Heliconia subgenus Heliconia

XML Treatment for Heliconia section Heliconia

XML Treatment for Heliconia section Lasia

XML Treatment for Heliconia section Cannastrum

XML Treatment for Heliconia section Longiflorae W.J.Kress, sect. nov.

XML Treatment for Heliconia section Lanea

XML Treatment for Heliconia section Griggsia

XML Treatment for Heliconia section Longae W.J.Kress, sect. nov.

XML Treatment for Heliconia section Episcopales

XML Treatment for Heliconia section Pastazae W.J.Kress, sect. nov.

## ﻿Additional information

### Author ORCIDs

W. John Kress https://orcid.org/0000-0002-0140-5267

Tomáš Fér https://orcid.org/0000-0002-0126-3684

Mónica M. Carlsen https://orcid.org/0000-0002-1663-0475

### Data availability

All of the data that support the findings of this study are available in the main text or Supplementary Information.

## Supplementary materials

Supplementary material 1 Specimen information, genomic sequence data, and accession numbers for the phylogenomic analysis Data type xlsx https://binary.pensoft.net/file/1218765This dataset is made available under the Open Database License (http://opendatacommons.org/licenses/odbl/1.0/). The Open Database License (ODbL) is a license agreement intended to allow users to freely share, modify, and use this Dataset while maintaining this same freedom for others, provided that the original source and author(s) are credited.W. John Kress, Tomáš Fér, Mónica M. Carlsen

Supplementary material 2 Full phylogeny of *Heliconia* with all species included in the genomic analysis with Local Posterior Probability (LPP) support values indicated Data type pdf Explanation note The six species with gray branches represent significant conflicts in genomic and morphological evidence, with latter given priority in species placement in the classification (see text). https://binary.pensoft.net/file/1218766This dataset is made available under the Open Database License (http://opendatacommons.org/licenses/odbl/1.0/). The Open Database License (ODbL) is a license agreement intended to allow users to freely share, modify, and use this Dataset while maintaining this same freedom for others, provided that the original source and author(s) are credited.W. John Kress, Tomáš Fér, Mónica M. Carlsen
