# Supplementary material for: ﻿Phylogenomics and a new classification of the tropical genus Heliconia L. (Monocots, Zingiberales, Heliconiaceae)
Source: PhytoKeys. 2025 Jan 13;251:37–66. doi: 10.3897/phytokeys.251.130409 (PMC11747779; doi:10.3897/phytokeys.251.130409)
Supplement: Supplementary material 2 — Full phylogeny of Heliconia with all species included in the genomic analysis with Local Posterior Probability (LPP) support values indicated [file phytokeys-251-037_article-130409__-s002.pdf]

Suppl. material 2

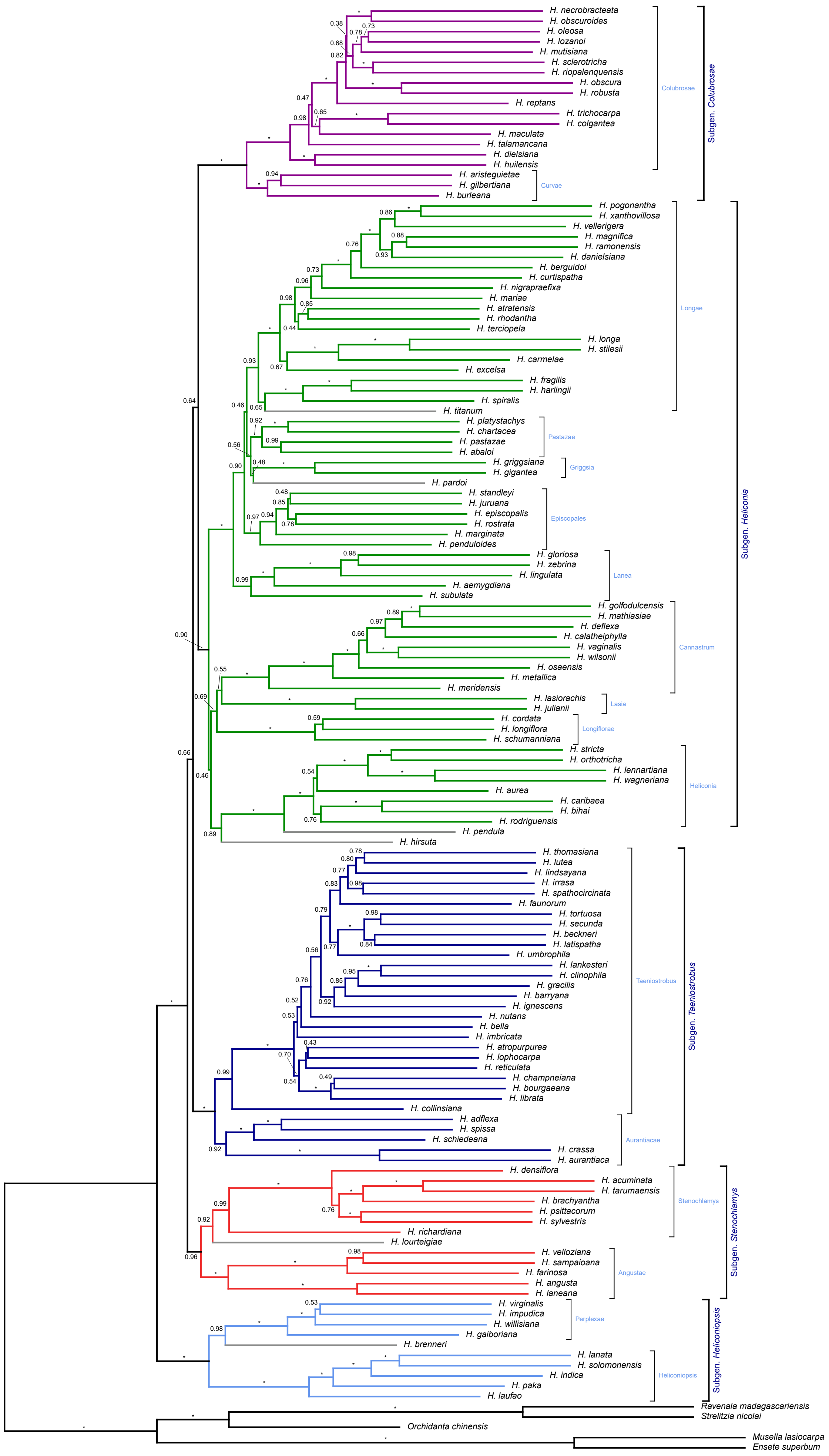

Figure S1. Full phylogeny of *Heliconia* with all species included in the genomic analysis with Local Posterior Probability (LPP) support values indicated. The six species with gray branches represent significant conflicts in genomic and morphological evidence, with latter given priority in species placement in the classification (see text).
